# “She gets out of control when she’s on her period”. Cyclic menstrual psychosis. A case report

**DOI:** 10.1192/j.eurpsy.2022.1084

**Published:** 2022-09-01

**Authors:** M.V. López Rodrigo, A. Osca Oliver, M. Palomo Monge, M.F. Tascón Guerra, M. Pérez Fominaya

**Affiliations:** 1 Hospital Nuestra Señora del Prado, Psiquiatría, Talavera de la Reina, Spain; 2 Hospital Nuestra Señora del Prado, Psiquiatria, Talavera de la Reina, Spain

**Keywords:** cyclic, menstruation, Psychosis, cyclic psychosis

## Abstract

**Introduction:**

Cyclical psychosis related to the menstrual cycle is an entity not included in the DSM-V and ICD-10 classifications, however there are data collected in the literature on cases that agree with this diagnosis. When reviewing cases, psychotic symptoms of sudden onset are described a few days before menstruation, with the symptoms resolving in a self-limited way when the bleeding ends. The end of psychotic symptoms is not directly related to the use of antipsychotics. The complete clinical picture is nonspecific and fluctuating. With acute onset, short duration, cyclical repetition, with psychotic symptoms (mutism, confusion, delusions, hallucinations) or a manic episode. We present the case of a 14-year-old adolescent with a history of epileptic seizures in childhood, without current treatment. She goes to the emergency department brought by her father and brother presenting psychomotor agitation, verbiage, flight of ideas, loss of the common thread in the speech, referring delusional ideas with experience of harm. His relatives report that he has not slept for a few days, with soliloquies, unmotivated laughter. They refer that the picture has been repeated in recent months during the days of menstruation.

**Objectives:**

Knowing a diagnosis not included in the current classifications.

**Methods:**

Imaging tests and neurological evaluations rule out organic picture.

**Results:**

Given the periodicity of the condition, the symptoms are self-limiting at the end of menstruation, without a clear relationship with psychopharmacological treatment (although agitation improves).

**Conclusions:**

Cyclical menstrual psychosis approximates affective disorders, especially bipolar disorder in adolescence. The role of psychotropic and hormonal treatment is debatable.

**Disclosure:**

No significant relationships.

